# Frontal Connectivity in EEG Gamma (30–45 Hz) Respond to Spinal Cord Stimulation in Minimally Conscious State Patients

**DOI:** 10.3389/fncel.2017.00177

**Published:** 2017-06-28

**Authors:** Yang Bai, Xiaoyu Xia, Zhenhu Liang, Yong Wang, Yi Yang, Jianghong He, Xiaoli Li

**Affiliations:** ^1^Institute of Electrical Engineering, Yanshan UniversityQinhuangdao, China; ^2^Department of Neurosurgery, PLA General HospitalBeijing, China; ^3^State Key Laboratory of Cognitive Neuroscience and Learning, Beijing Normal UniversityBeijing, China; ^4^IDG/McGovern Institute for Brain Research, Beijing Normal UniversityBeijing, China

**Keywords:** spinal cord stimulation, EEG, minimally conscious state, gamma, functional connectivity

## Abstract

Spinal cord stimulation (SCS) has become a valuable brain-intervention technique used to rehabilitate patients with disorders of consciousness (DOC). To explore how the SCS affects the cerebral cortex and what possible electrophysiological mechanism of SCS effects on the cortex, the present study investigated the functional connectivity and network properties during SCS in minimally conscious state (MCS) patients. MCS patients received both SCS and sham sessions. Functional connectivity of the phase lock value (PLV) in the gamma band (30–45 Hz) was investigated at the pre-, on- and post-SCS stages. In addition, to evaluate global network properties, complex network parameters, including average path length, cluster coefficient and small-world, were measured. When SCS was turned on, significantly decreased connectivity was noted in the local scale of the frontal-frontal region and in the large scales of the frontal-parietal and frontal-occipital regions. The global network showed fewer small-world properties, average path lengths increased and cluster coefficients decreased. When SCS was turned off, the large-scale connectivity and global network returned to its pre-SCS level, but the local scale of frontal-frontal connectivity remained significantly lower than its pre-SCS level. Sham sessions produced no significant changes in either functional connectivity or network. The findings directly showed that SCS could effectively intervene cortical gamma activity, and the intervention included immediate global effects (large scale connectivity and network alteration only occurred in stimulation period) and long-lasting local effects (local scale connectivity alteration persist beyond stimulation period). Moreover, considering the mechanism and propagation of gamma activity, it indicates that the frontal cortex plays a crucial role in the SCS effects on the cerebral cortex.

## Introduction

Despite considerable research, there is currently no effective standardized treatment for patients with disorders of consciousness (DOC). However, in recent years, spinal cord stimulation (SCS) has become a valuable brain-intervention technique for rehabilitating DOC patients because it is a less-invasive, simpler surgical procedure than deep-brain stimulation (Georgiopoulos et al., 2010; Mattogno et al., 2017). Studies have shown its efficacy in modulating the brains of DOC patients (Yamamoto et al., 2012, 2013, 2017), but the underlying mechanism of its effects on the cerebral cortex is still unclear.

Electrophysiological studies investigating the effects of SCS on DOC patients are limited. Studies using pain-related P250 amplitude have shown that SCS may indirectly stimulate the frontal cortex, which is part of the awareness and attention network (Yampolsky et al., 2012; Yamamoto et al., 2013). In a previous study, we reported that SCS modulated frontal delta and gamma activity in patients in a minimally conscious state (MCS) (Bai et al., 2017), showing that SCS modulated the brain functions of MCS patients and providing EEG evidence supporting the potential mechanism for doing so: stimulating the reticular formation and further affecting the frontal cortical region through the formation-thalamus-cortex network. However, further research is needed to provide detailed evidence about the pathway by which SCS affects the cerebral cortices of DOC patients.

Gamma oscillatory activity is thought to be a fundamental mechanism that integrates neural networks within and across brain structures, facilitates coherent sensory registration and mediates cognitive functions (Kaiser and Lutzenberger, 2005; Herrmann et al., 2010; Roye et al., 2010). This activity may represent a coding operator for brain functions and inter-area communications (Basar, 2013). Gamma connectivity has been implicated relevant in subjective consciousness (Llinas et al., 1998; Singer, 1998), and gamma coherence has been shown to be a fundamental prerequisite in the aware process and the potential to participate cognitive processes (Naro et al., 2016). In addition, gamma connectivity has been identified as an important awareness-level marker in DOC patients (Cavinato et al., 2015; Naro et al., 2016). In MCS and vegetative state (VS) patients, significant differences have been demonstrated in the responses of short-range parietal and long-range fronto-parietal gamma coherences to simple sensory stimulus modalities (Cavinato et al., 2015). Our previous study found that SCS, particularly at 70 Hz, significantly changed gamma activity, increasing its relative power and decreasing both coherence and global synchronization. Therefore, considering gamma activity's critical role in cognitive and brain function, gamma activity alteration induced by SCS may increase understanding of the possible physiological basis for the effects of SCS.

Functional connectivity and corresponding network parameters have been identified as valuable characteristic to assess the brain states of DOC patients (Kotchoubey et al., 2013; Chennu et al., 2014; Monti et al., 2015). Functional connectivity measures the relationships among the activities measured in various regions of interest in the brain. Analysing complex networks based on graph theory has enabled the application of new methods useful to investigating both local and global properties of functional connectivity in the brain (Bullmore and Sporns, 2009). Combination of functional connectivity and network properties play a prominent role in analysing, describing and understanding human brain function (Stam and Reijneveld, 2007). Therefore, the present study focused on assessing the functional connectivity and network characteristics of gamma activity. Given that SCS at a frequency of 70 Hz proved to significantly change gamma activity, 70 Hz was chosen as the frequency parameter of stimulation during sessions that included EEG recording at the pre-, on- and post-SCS stages. We hypothesized that the relationships among various brain regions would identify the pathway responsible for the SCS effects.

## Experimental procedures

### Patients

As reported in our previous study, 16 MCS patients, aged 19–65, who had received SCS implants were enrolled in a multiple-session stimulation study. All participants were at least 3 weeks post-surgery and had stable clinical states. During the study, 5 participants dropped out due to infection and other clinical factors. Table 1 shows the clinical features of the 11 participants who completed the entire study. The consciousness state of each patient was assessed using the JFK Coma Recovery Scale-Revised (JFK CRS-R) (Giacino et al., 2004). Any treatment or drugs that could modify cortical excitability were excluded. Written informed consent to participate in the study was obtained from the patient's caregivers, and the study was approved by the ethics committee of PLA Army General Hospital.

**Table 1:** Participants' demographic characteristics.

**Patient**	**Etiology**	**Post-injury (months)**	**CRS-R**
Patient 1	Hemorrhage	18	8
Patient 2	Hemorrhage	12	10
Patient 3	Hemorrhage	11	7
Patient 4	Hemorrhage	11	7
Patient 5	Anoxia	4	10
Patient 6	Hemorrhage	3	9
Patient 7	Hemorrhage	4	7
Patient 8	Anoxia	3	7
Patient 9	Infarction	4	7
Patient 10	Anoxia	8	7
Patient 11	Anoxia	28	9

*CRS-R, Coma recovery scale-revised*.

### Stimulation protocol

The participants received both sessions that included real stimulation and sham stimulations, with at least 2 days' washout between sessions. Stimulation was delivered by a pulse generator (Prime Advanced, Medtronic Inc., Minneapolis, MN, USA) that delivered electric pulses with a 3-V amplitude and a 210-μs pulse width. The pulse generator was implanted under the anterior chest wall. And the pulse generator was connected to stimulation electrodes through Touhy needle. The needle was implanted into the midline epidural space at the cervical-thoracic junction. And the stimulation electrodes were inserted into the epidural space of the cervical vertebrae, and placed at the C2–C4 levels. The stimulation was caused by periodic occurrence of voltage difference between two electrodes. The stimulation parameters could be setup by a wireless controller *in vitro*. In this study, the stimulation frequency parameter was set at 70 Hz and delivered for 20 min consecutively. Sham stimulations were conducted with the stimulator turned off.

### EEG recordings and pre-processing

Resting state EEG was recorded for 10 min before stimulation, for 20 min during the real or sham stimulation and for 10 min immediately after the stimulation. EEGs were recorded using 32 channels (BrainAmp 64 MRplus, Brain Products, Germany) and Ag/AgCl pin electrodes having a sampling rate of 1 kHz. The skin/electrode impedance was maintained below 5 kΩ. During the experiments, participants were behaviorally awake (eyes open, EO), and if they showed signs of sleepiness (prolonged eye closure, EC), either the JFK CRS-R arousal-facilitation protocol was applied or the experiment was suspended.

EEG pre-processing was conducted using EEGLAB software version 12.0.2.5b, running on a MATLAB environment (Version 2013b, MathWorks Inc.; Natick, MA, USA). The 50-Hz power signal was removed by a notch filter. The EEG signal was band filtered between 1 and 45 Hz. The Independent Component Analysis (ICA) was used to identify and remove artifact-relevant components, including eye movements and muscle activation. The EEG data were divided into epochs of 10 s with overlaps of 50%. The selected artifact-free epochs were average referenced.

### EEG analysis

#### Phase locking value (PLV)

Connectivity between pairwise channels in the gamma band (30–45 Hz) was computed using phase synchronization, which is briefly described as follows. For each epoch EEG signal, the instantaneous phases φ_*x*_(*t*) and φ_*y*_(*t*) of the pairwise channel were evaluated based on the Hilbert transform. Then, the phase difference was defined by

(1) $$\begin{array}{r} \Delta \phi_{xy}\left(t\right)=\varphi_{x}\left(t\right)-\varphi_{y}\left(t\right). \end{array}$$

Several indices based on the phase difference within a short term can be used to indicate the phase synchronization between two series (Rosenblum et al., 2001). The present study applied PLV based on the circular variance of the phase difference, yielding

(2) $$\begin{array}{r} {PLV}_{xy}=\frac{1}{N}|\sum_{t=1}^{N}e^{j\Delta \varphi_{xy}\left(t\right)}|. \end{array}$$

This measure of PLV varied between 0 and 1, and the computation involved no parameter choices. Therefore, the synchrony can be described by a phase-synchronization matrix *C* with each element of *PLV*_*xy*_.

#### Graph theoretical analysis

Graph theoretical analysis was performed on the phase synchronization matrices. The nodes in the graph were defined as the electrodes and the links as the measure of the phase synchronization between the nodes. Weighted graphs were created using synchronization matrix *C* with each element of *PLV*_*xy*_.

Graphs can be characterized using various measures, two of the most fundamental of which are the clustering coefficient, which denotes the likelihood that neighbors of a vertex will also be connected to each other, and the average path length, which indicates the average number of edges of the shortest path between pairs of vertices. Stam et al. (2009) provided full definitions for calculating the clustering index (*C*_*w*_) and the path length (*L*_*w*_) to analyse weighted networks. To calculate the clustering index from weighted networks, the weights between the node *i* and the other nodes *j* should be symmetrical (ω_*ij*_ = ω_*ji*_), and 0 ≤ ω_*ij*_ ≤ 1, as proposed by Onnela et al. (2005). Indeed, both conditions are readily fulfilled when using PLV values as the weight definition. Then, the weighted clustering index of vertex *i* is defined as

(3) $$\begin{array}{r} C_{i}=\frac{\sum_{k\neq i}\sum_{l\neq i,l\neq k}\omega_{ik}\omega_{il}\omega_{kl}}{\sum_{k\neq i}\sum_{l\neq i,l\neq k}\omega_{ik}\omega_{il}}. \end{array}$$

Note that all sums terms with *k* = *i*, *l* = *i* or *k* = *l* are skipped. The mean clustering of the total network is defined as

(4) $$\begin{array}{r} C_{w}=\frac{1}{N}\overset{N}{\underset{i=1}{\sum }}C_{i} \end{array}$$

Then, the length of a weighted path between two vertices is defined as the sum of the lengths of the edges of this path. The shortest path *L*_*ij*_ between two vertices *i* and *j* is the path between *i* and *j* with the shortest length. The averaged path length of the entire network is computed as

(5) $$\begin{array}{r} L_{w}=\frac{1}{\left(1/N\left(N-1\right)\right)\sum_{i=1}^{N}\sum_{j\neq i}^{N}\left(1/L_{ij}\right)} \end{array}$$

In this formula, the harmonic mean is used to handle disconnected edges resulting in infinite path lengths (i.e., 1/∞ → 0) (Newman, 2003). Then, the small-world parameter can be calculated as $S=\frac{C_{w}}{L_{w}}$.

To obtain measures that are independent of network size, the mean edge weight and the mean path length were compared to the mean of 50 random networks. The $C_{w}^{s}$ and $L_{w}^{s}$ denote weighted clustering coefficient and path length, respectively, averaged over an ensemble of 50 random, surrogate networks that were derived from the original network by randomly reshuffling the edge weights. Then, the final index was obtained from ${\hat{C}}_{w}=C_{w}/C_{w}^{s}$ and ${\hat{L}}_{w}=L_{w}/L_{w}^{s}$. Finally, the small-world parameter was denoted as $S=\frac{{\hat{C}}_{w}}{{\hat{L}}_{w}}$.

## Results

**Figure 1:**
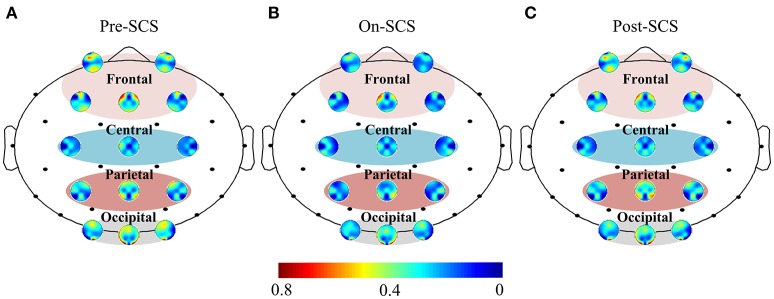
Functional connectivity of electrodes in regions of interest and overall brain in gamma band. **(A)** Average functional connectivity of the electrodes with overall brain pre-SCS. **(B)** Average functional connectivity of the electrodes with overall brain on-SCS. **(C)** Average functional connectivity of the electrodes with others post-SCS. Each colored circle shows the connectivity between the identified electrode and other electrodes. The regions of interest are the frontal, central, parietal and occipital. Colors indicate the average strength of connectivity.

Functional connectivity was measured using the PLV between each set of pairwise EEG channels. Then, interest regions were defined as follows: frontal (Fp1, Fp2, Fz, F3, and F4), central (Cz, C3, and C4), parietal (Pz, P3, and P4) and occipital (Oz, O3, and O4). Figure 1 shows the functional connectivity of electrodes in the regions of interest and the overall brain in gamma band. Comparison of on-SCS and pre-SCS shows a distinct decrease of connectivity among the frontal, frontal-parietal and frontal-occipital regions. After the SCS was turned off, connectivity of frontal-frontal, frontal-parietal and frontal-occipital were markedly returned.

**Figure 2:**
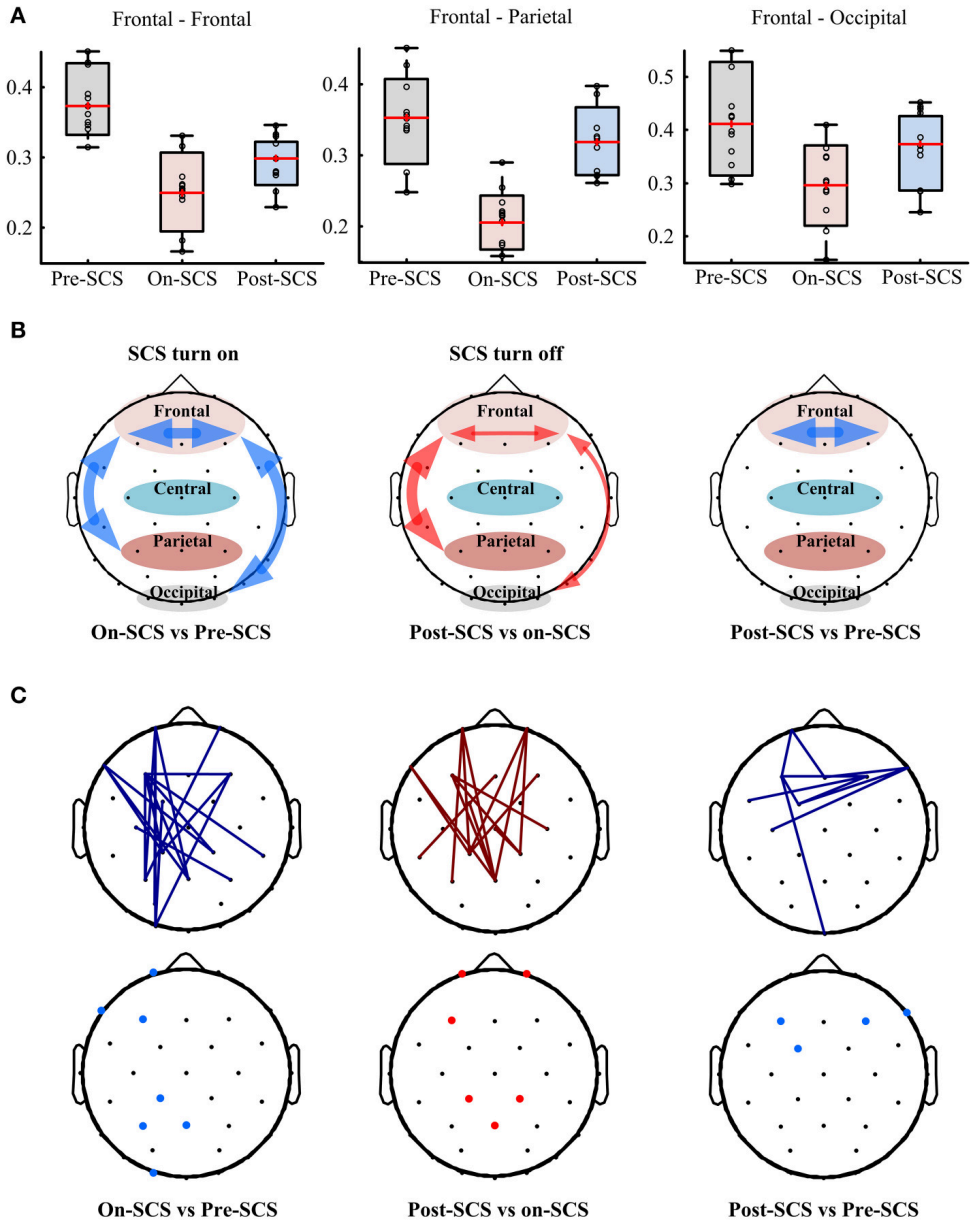
**(A)** Functional connectivity in patients' gamma bands at pre-, on- and post-SCS. **(B)** Compasions of patients' average frontal-frontal, frontal-parietal and frontal-occipital connectivity using paired *t*-test. Heavy red arrows mean significant increases, thin red arrows mean increase without significance, and blue arrows mean significant decreases. **(C)** Top panel shows significantly altered connectivities and bottom panel shows critical electrodes compared between pairwise stages. Red lines mean significantly increased connectivity, and blue lines mean significant decreased connectivity. Red dots mean critical electrodes included in significantly increased connectivities, and blue dots mean critical electrodes included in significantly decreased connectivities.

SCS induced connectivity changes were found in frontal-frontal, frontal-parietal, and frontal-occipital (Figure 2A). To investigate the changes at different stages, we compared the average connectivity between pairwise stages using paired *t*-tests: On-SCS vs. Pre-SCS, Post-SCS vs. On-SCS and Post-SCS vs. Pre-SCS (Figure 2B). Bonferroni correction was performed after multiple comparisons. When SCS was turned on (On-SCS vs. Pre-SCS), significant decreases of connectivity were found in frontal-frontal (*p* < 0.001), frontal-parietal (*p* < 0.001) and frontal-occipital (*p* = 0.011). When SCS was turned off, all the connectivity rebounded but only with significance in the frontal-parietal (*p* < 0.001). But when post-SCS compared with pre-SCS, significance was only found within frontal-frontal connectivity (*p* < 0.001). As Table 2 shows, in sham sessions, in all three stages, the average connectivity showed no significant changes.

**Table 2:** Gamma band functional connectivity and network parameters (mean ± standard deviation) pre-SCS, on-SCS and post-SCS in sham sessions.

	**Pre-SCS**	**On-SCS**	**Post-SCS**
Frontal-Frontal	0.374 ± 0.047	0.380 ± 0.042	0.357 ± 0.031
Frontal-Parietal	0.341 ± 0.052	0.358 ± 0.070	0.332 ± 0.060
Frontal-Occipital	0.399 ± 0.076	0.389 ± 0.076	0.386 ± 0.073
Average path length	0.189 ± 0.020	0.186 ± 0.018	0.190 ± 0.013
Cluster coefficient	5.664 ± 0.396	5.584 ± 0.458	5.627 ± 0.048
Small-World	31.26 ± 5.448	31.24 ± 6.784	31.29 ± 3.806

Then, pairwise comparisons of each connectivity were performed using paired *t*-tests, and correction of the false discovery rate was performed after multiple comparisons (*q*-value = 0.05). Top panel of Figure 2C shows the significantly altered connecitities in comparison of pairwise stages. Then, we defined the electrodes which were included in at least three significantly changed connectivities as critical electrodes (bottom panel of Figure 2C). The location of the significantly altered connectivity and critical eletrodes was generally consistent with the average connectivity findings. When SCS was turned on or turned off, the significantly altered connectivity and critical electrodes were both located mostly in the frontal and parietal regions. And after SCS stimulation, the locations of the significantly decreased connectivity and the critical electrodes were found in frontal regions.

**Figure 3:**
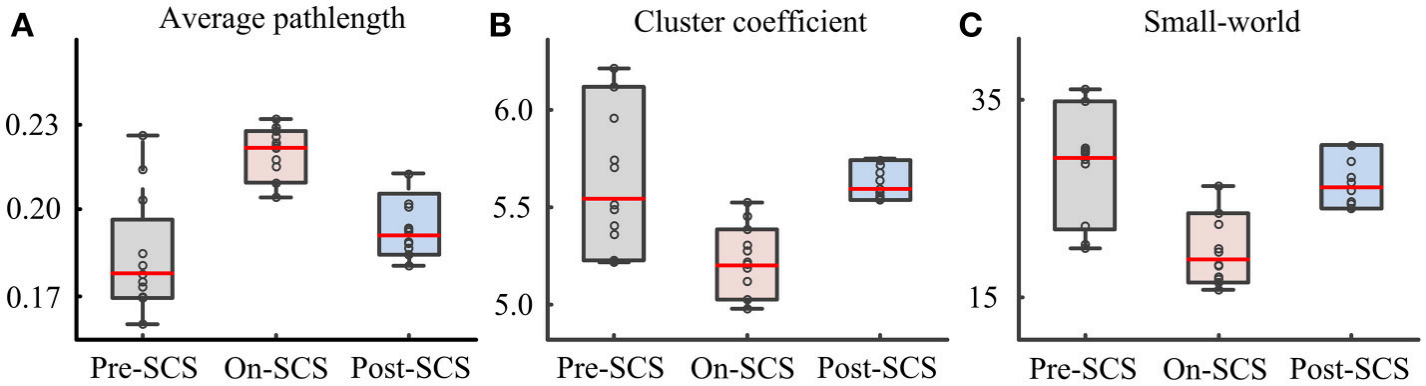
Network parameters in gamma bands at pre-SCS, on-SCS and post-SCS. **(A)** Average path length. **(B)** Cluster coefficients. **(C)** Small-world.

In order to explore the effects of SCS on the global cerebral cortex, graph theoretical based network parameters were calculated using the connectivity matrix in the gamma band at three stages: path length, cluster coefficient and small-world. Comparisons between pairwise stages were performed using paired *t*-tests with Bonferroni corrections. Figure 3 shows the boxplot of the parameters at each stage. Significant increases of average path length (*p* = 0.004) and decreases of cluster coefficient (*p* = 0.040) were found during SCS stimulation period. The global network showed less small-world property. And the network perturbation company with the stimulation. When SCS was turned off, the network parameters rebound to the baseline level. In sham sessions, the various stages produced no significant alterations of the network parameters.

## Discussion

SCS has been demonstrated as a valuable brain-intervention technique for rehabilitating DOC patients (Georgiopoulos et al., 2010; Mattogno et al., 2017; Yamamoto et al., 2017). But there was still few studies investigating the potential mechanism. In a previous study (Bai et al., 2017), we reported that SCS at a frequency of 70 Hz could effectively modulate MCS patients' frontal gamma activities by increasing the relative power and decreasing the coherence and global synchronization. In order to further explore the effects of SCS with 70 Hz on interventing gamma activity, the present study measured functional connectivity and network properties in the gamma band at different SCS stages. By this way, we aim to investigate how the SCS effects on the cortex and what the potential pathway participate SCS modulation on the cerebral. Comparing with pre-SCS, SCS turning on induced significant decreases in functional connectivity in local scale of frontal-frontal and large scale of frontal-parietal and frontal-occipital regions. When SCS was turned off, the large scale connectivity and global network features returned to the pre-SCS level. But the local scale of frontal-frontal connectivity stays lower than pre-SCS. The findings directly show that, SCS turning on effectively modulate the gamma activity. The interventaion includes immediate global effects (large scale connectivity and network property), and local effects (local scale connectivity) which last beyond period of the stimulation. The global and local effects imply that regions of frontal, parietal and occipital were the direct targets of SCS. But, potential after-effects of local or global effects of SCS on consciousness rehabilitation should be further explored in long-term clinical assessment.

These results provide further evidence supporting the critical role of the frontal cortex in the SCS effects. All significantly altered connectivity was relevant to the frontal region, no matter whether it was in global or local effects. When SCS was turned off, connectivity of the frontal-parietal and frontal-occipital returned to pre-SCS levels, but frontal-frontal connectivity in post-SCS remained lower than pre-SCS. These decreases in the frontal regions were consistent with the coherence results from our previous study (Bai et al., 2017). Evidence demonstrating the role of the frontal cortex in SCS has also been provided by other studies using pain-related P250 amplitude. These studies demonstrated the capability of SCS to directly affect the frontal cortex (Yampolsky et al., 2012; Yamamoto et al., 2013). Therefore, the intervention global effects occurring in the parietal and occipital cortices may be induced by their interaction with frontal cortex, and the interaction could be represented in gamma connectivity.

Considering the crucial role of gamma activity in brain function, the gamma alteration induced by SCS stimulation may imply brain modulation in DOC patients. Gamma activity can be measured in a wide range of cortical and subcortical structures (Basar and Bullock, 1992). One important potential mechanism considered existing a subcortical pacemaker (thalamus) that drove the cortex at a frequency in the gamma band (Llinas et al., 1998). Then, the gamma activity measured in the cortex was propagated by thalamo-cortical connections (Steriade et al., 1996; Llinas et al., 2005), meaning that the intra-laminar thalamic nuclei might drive large areas of the cortex using gamma oscillations (Llinas and Ribary, 1993). Therefore, these global changes in connectivity and network in the gamma activity suggest the capability of SCS to modulate the gamma pacemaker: thalamus. The altered thalamus interrupts the established model of gamma “command” activity in the cortex, thus causing fewer small-world network features, including increasing average path length and decreasing cluster coefficient, in the cortex. When combined with the results of our previous research, those of the present study suggest that gamma activity detected in the cortex, especially the frontal cortex, may be a significant biomarker for evaluating the effects of SCS.

The results of the present study provide EEG evidence demonstrating the anatomical pathway by which SCS modulates the brain: the thalamus-cortex connection. This is consistent with the indications that the SCS mechanism may be that it enhances the specific firing of the cerebral cortex by exerting a direct effect on the reticular formation-thalamus pathway. In addition, the crucial role of the frontal region in altering connectivity along with SCS stimulation prompts us to propose the frontal cortex as a relay station in the SCS modulation pathway. SCS affects the cerebral cortex by first modulating the frontal cortex and then propagating to other regions via frontal cortex connectivity. Coincidentally, this pathway has been included in a meso-circuit model that attempts to explain DOC after brain injuries (Schiff, 2010). In this explanation, the connections between the thalamus, frontal cortex and parietal/occipital/temporal cortices are crucial to maintaining the consciousness-related network (Giacino et al., 2014). The significant changes in connectivity in the gamma that are induced by SCS being turned on and off are closely related to the three important nodes (thalamus, frontal cortex and parietal/occipital cortices) in this circuit-level pathway. Therefore, we propose that the effects of SCS on consciousness may benefit from interfering with the circuit-level network.

## Conclusions

The present study investigated connectivity and network characteristics in the gamma band at pre-, on- and post-SCS using an SCS frequency of 70 Hz. Significantly frontal related connectivity alterations and network parameters changes were found in gamma band. The findings indicated that SCS could effectively affect the cerebral cortex and the intervention includes immediate global effects (large scale connectivity of frontal-parietal and frontal-occipital, and network alteration) and long-lasting local effects (local connectivity of frontal-frontal). Considering the mechanism of gamma generation and propagation, we suggest that the frontal cortex plays the crucial role in pathway of the stimulation: the SCS alters the frontal cortex by using the thalamus-cortex connection, and then the global brain are modulated via connectivity with the frontal cortex. In addition, the pathway may be the anatomical basis by which the SCS modulates the brain. Since the thalamus, frontal cortex and parietal/occipital cortex play crucial roles in the consciousness-related network, the effects of SCS on consciousness may benefit from interfering with the circuit-level network.

## Ethics statement

This study was carried out in accordance with the recommendations of the ethics committee of PLA Army General Hospital with written informed consent from all subjects. All subjects gave written informed consent in accordance with the Declaration of Helsinki. The protocol was approved by the ethics committee of PLA Army General Hospital.

## Author contributions

YB had full access to all the data in the study and takes responsibility for the integrity of the data and the accuracy of the data analysis. Study concept and design: YB and XX. Acquisition, analysis, or interpretation of data: YB and XX. Drafting of the manuscript: YB. Critical revision of the manuscript for important intellectual content: All authors. Statistical analysis: YB. Obtained funding: JH and XL. Administrative, technical, or material support: YW. Study supervision: JH and XL.

### Conflict of interest statement

The authors declare that the research was conducted in the absence of any commercial or financial relationships that could be construed as a potential conflict of interest.
