# Corrigendum: Frontal Connectivity in EEG Gamma (30–45 Hz) Respond to Spinal Cord Stimulation in Minimally Conscious State Patients

**DOI:** 10.3389/fncel.2017.00251

**Published:** 2017-08-18

**Authors:** Yang Bai, Xiaoyu Xia, Zhenhu Liang, Yong Wang, Yi Yang, Jianghong He, Xiaoli Li

**Affiliations:** ^1^Institute of Electrical Engineering, Yanshan University Qinhuangdao, China; ^2^Department of Neurosurgery, PLA Army General Hospital Beijing, China; ^3^State Key Laboratory of Cognitive Neuroscience and Learning, Beijing Normal University Beijing, China; ^4^IDG/McGovern Institute for Brain Research, Beijing Normal University Beijing, China

**Keywords:** spinal cord stimulation, EEG, minimally conscious state, gamma, functional connectivity

In the published article, there was an error in the Affiliation 2. Instead of “Department of Neurosurgery, PLA General Hospital, Beijing, China,” it should be “Department of Neurosurgery, PLA Army General Hospital, Beijing, China.” The authors apologize for this error and state that this does not change the scientific conclusions of the article in any way.

## Conflict of interest statement

The authors declare that the research was conducted in the absence of any commercial or financial relationships that could be construed as a potential conflict of interest.

